# E3 Ubiquitin Ligase Nedd4 Promotes Japanese Encephalitis Virus Replication by Suppressing Autophagy in Human Neuroblastoma Cells

**DOI:** 10.1038/srep45375

**Published:** 2017-03-28

**Authors:** Qingqiang Xu, Naiwei Zhu, Shenglin Chen, Ping Zhao, Hao Ren, Shiying Zhu, Hailin Tang, Yongzhe Zhu, Zhongtian Qi

**Affiliations:** 1 Department of Microbiology, Second Military Medical University, Shanghai Key Laboratory of Medical Biodefense, Shanghai 200433, China; 2 Department of Chemical Defense Medicine, Second Military Medical University, Shanghai 200433, China; 3 General Hospital of the Tibet Military Area Command, Tibet 850007, China

## Abstract

Japanese encephalitis virus (JEV) is a mosquito-borne flavivirus that causes the most prevalent viral encephalitis in Asia. Since JEV is a neurotropic virus, it is important to identify key molecules that mediate JEV infection in neuronal cells and to investigate their underlying mechanisms. In this study, the critical role of Nedd4, an E3 ubiquitin ligase that is highly expressed in the central nervous system, was examined in JEV propagation. In SK-N-SH neuroblastoma cells, Nedd4 was up-regulated in response to JEV infection. Moreover, down-regulation of Nedd4 resulted in a significant decrease in JEV replication without alterations in virus attachment and internalization or in JEV pseudotyped virus infection, suggesting that Nedd4 participates in the replication but not in the entry stage of JEV infection. Further functional analysis showed that Nedd4 attenuated JEV-induced autophagy, which negatively regulates virus replication during infection. These results suggest that Nedd4 facilitates the replication of JEV by suppressing virus-induced autophagy. Taken together, our results indicate that Nedd4 plays a crucial role in JEV infection of neuronal cells, which provides a potential target for the development of novel treatment to combat JEV infection.

Japanese encephalitis (JE) is a serious mosquito-borne viral encephalitis disease caused by infection with Japanese encephalitis virus (JEV)^1^. JEV belongs to the JEV serogroup in the genus *Flavivirus*, family *Flaviviridae*, and has a single-stranded, positive-sense RNA genome ~11 kb in length^2^. JEV is a neurotropic virus, and infection causes an acute central nervous system (CNS) inflammatory disease. JEV-induced viral encephalitis is characterized by profound neuronal cell damage and extensive inflammation in the CNS^3^,^4^. During infection, neurons can directly undergo apoptosis or necrosis due to viral multiplication or through a bystander mechanism in which overactivation of microglia/glial cells leads to indirect neuronal killing via the secretion of numerous proinflammatory cytokines^5^. Infection of the CNS with JEV causes viral encephalitis; however, the molecular pathogenesis that occurs as a result of JEV infection of neurons remains unclear, and specific treatment to inhibit JEV-induced neuroinflammation is not available. Therefore, the discovery of cellular molecules that mediate JEV infection of neuronal cells is important.

Viruses utilize the function of cellular factors to establish an effective infection. Mounting evidence has highlighted the essential regulatory role of the ubiquitin-proteasome system (UPS) proteins in host-virus interactions^6^,^7^. Functional UPS proteins are essential for the replication of major human pathogens such as herpesviruses, adenoviruses, poxviruses, hepadnaviruses, and influenza viruses, among others^8^,^9^. Some viruses encode their own E3 ubiquitin ligases that target unwanted cellular proteins for degradation, such as infected cell polypeptide 0 (ICP0) of herpes simplex virus type 1 (HSV-1), a multifunctional protein with RING domain E3 ubiquitin ligase activity^10^. Moreover, ubiquitination also plays an important role in viral evasion of host innate immunity^9^. Ubiquitin-enabled interference of viruses by the innate immune response has emerged as a central immune evasion mechanism in almost every viral species studied^11^.

Nedd4 (neuronal precursor cell-expressed, developmentally downregulated 4) is an E3 ubiquitin ligase with a catalytic domain of the HECT class. Nedd4 was originally identified as a developmentally regulated gene and is highly expressed in the mouse embryonic CNS^12^. Nedd4 has been shown to play important physiological roles in regulating almost every facet of neuronal function, from development to neurotransmission and synaptic plasticity^13^. It has been identified as an important regulator of dendrite formation and arborization in both hippocampal and cortical neurons^14^,^15^. Moreover, Nedd4-induced ubiquitination and subsequent down-regulation of PTEN mediates the neuronal response to zinc-induced damage of the CNS^16^. In mammalian cells, Nedd4 participates in diverse cellular processes, including the regulation of the tumour suppressors^17^ and protein trafficking^18^. Recently, Nedd4 has been demonstrated to interfere with autophagy^19^,^20^. Furthermore, Nedd4 also plays an important role in the retroviral budding process by ubiquitination of Gag polyproteins^12^, suggesting that Nedd4 participates in virus infection. However, the role of Nedd4 in neurotropic virus infection is unclear.

In this study, JEV infection induced elevated expression of Nedd4 in human SK-N-SH neuroblastoma cells. Down-regulation of Nedd4 resulted in a significant inhibition of JEV infection. Further functional analysis showed that JEV used Nedd4 to facilitate its replication by suppressing viral-induced autophagy. Our results indicate the crucial role of Nedd4 in JEV infection of neuronal cells, and provide a potential target for developing novel antiviral strategies to combat JEV.

## Results

### JEV infection up-regulates the expression of Nedd4 in SK-N-SH cells

**Figure 1:**
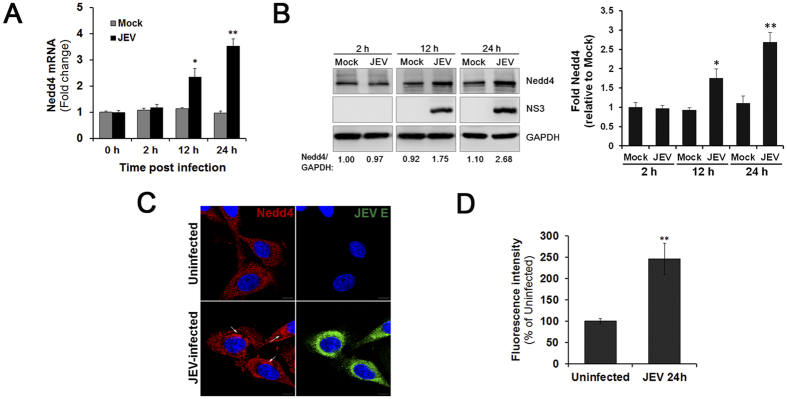
Nedd4 expression is up-regulated in SK-N-SH cells during JEV infection. SK-N-SH cells were infected with JEV at an MOI of 1.0. The cells were collected at different time points. (**A**) Nedd4 mRNA levels were determined by RT-qPCR at different time points p.i. (**B**) The protein levels of Nedd4 and JEV NS3 were examined in mock- and JEV-infected SK-N-SH cells via Western blot using the indicated antibodies. GAPDH served as a control for equal sample loading. The level of Nedd4 was quantitated by densitometric analysis using ImageJ software and normalized to GAPDH. (**C**) Localization of Nedd4. SK-N-SH cells were fixed at 24 h p.i. and were subjected to immunofluorescence to detect JEV E (green) and Nedd4 (red). The nuclei were stained with DAPI (blue). Images were obtained using a confocal microscope (Scale bar = 10 μm). (**D**) The fluorescence intensity of Nedd4 was processed and quantified using ImageJ software. The data are shown as the mean ± SD of three independent experiments. *P < 0.05; **P < 0.01.

The human SK-N-SH neuroblastoma cell line was used in this study because of its similarity to primary neuronal cells^21^. This cell line has previously been widely used to study neurotropic viruses. To determine whether JEV infection alters Nedd4 expression, SK-N-SH cells were infected with JEV at a multiplicity of infection (MOI) of 1.0, and Nedd4 mRNA levels were then determined by quantitative real time PCR (RT-qPCR) at different time points post infection (p.i.). As shown in Fig. 1A, Nedd4 mRNA levels were up-regulated after JEV infection. Moreover, Nedd4 protein levels were examined in mock- and JEV-infected SK-N-SH cells by Western blot (Fig. 1B). Infection was confirmed by detection of the viral nonstructural NS3 protein. Although the levels of GAPDH were similar in infected and mock-infected cells, Nedd4 levels were significantly increased in JEV-infected cells by 12 h p.i. and 24 h p.i. (Fig. 1B). To determine whether JEV infection alters Nedd4 localization, we performed immunofluorescent staining. As shown in Fig. 1C, Nedd4 staining was more apparent in the perinuclear region in JEV-infected cells compared with uninfected cells when examined at 24 h p.i. An increase in Nedd4 fluorescence intensity was also observed in infected cells compared with uninfected cells when examined at 24 h p.i. (Fig. 1D). These data indicate that JEV infection up-regulates the expression of Nedd4 in SK-N-SH cells and promotes the aggregation of Nedd4 in the perinuclear region of the cell.

### Nedd4 knockdown inhibits JEV infection

**Figure 2:**
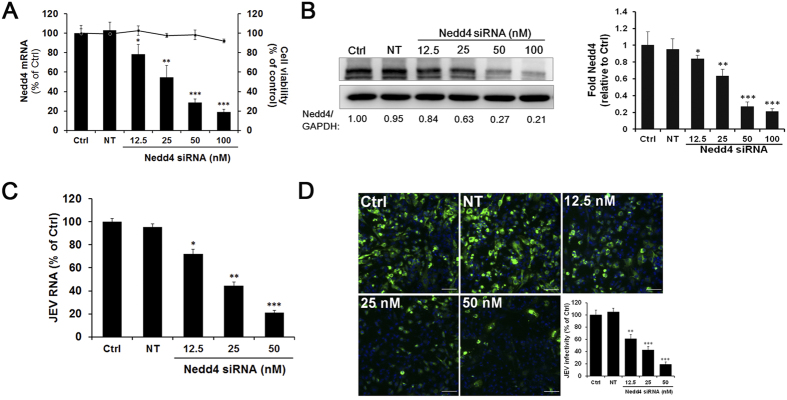
Nedd4 knockdown inhibits JEV infection. SK-N-SH cells were transfected with Nedd4-specific siRNA (12.5, 25, 50, 100 nM) or the Non-Target (NT) siRNA (100 nM) for 72 h. (**A**) The reduction of Nedd4 after treatment with different concentrations of siRNA was measured by RT-qPCR. An MTT assay was used to assess the siNedd4-induced cytotoxic effect. (**B**) The level of Nedd4 protein was detected by Western blot, quantitated by densitometric analysis using ImageJ software and normalized to GAPDH. (**C**) Cells transfected with Nedd4 siRNA or the NT siRNA (50 nM) were infected with JEV (MOI = 1.0). JEV infection was assessed by monitoring intracellular JEV RNA at 24 h p.i. by RT-qPCR. (D) SK-N-SH cells were fixed at 24 h p.i. and were subjected to immunofluorescence to detect JEV E (green). The nuclei were stained with DAPI (blue). Images were obtained using a confocal microscope and quantitated using ImageJ software (Scale bar = 50 μm). The data represent the mean ± SD of three independent experiments. *, P < 0.05; **, P < 0.01; ***, P < 0.001.

We next assessed the effect of Nedd4 knockdown on JEV infection. SK-N-SH cells were transfected with a Nedd4-specific siRNA or a Non-Target (NT) siRNA and then infected with JEV (MOI = 1.0) at 72 h posttransfection. As shown in Fig. 2A, the transfection of cells with the siRNA targeting Nedd4 caused a dose-dependent reduction in the Nedd4 mRNA level when compared with the level in the NT siRNA-transfected cells. Minimal cellular cytotoxicity was observed in the Nedd4 siRNA-treated cells at all concentrations (Fig. 2A). Depletion of Nedd4 in SK-N-SH cells was confirmed by Western blot (Fig. 2B). JEV infection was assessed by monitoring intracellular JEV RNA by RT-qPCR. The data showed a dose-dependent inhibition of JEV infection with increasing concentrations of Nedd4 siRNA (Fig. 2C). Moreover, Nedd4 knockdown also resulted in a dose-dependent decrease in the number of JEV E protein-positive cells at 24 h p.i. (Fig. 2D), further demonstrating the important role of Nedd4 in JEV infection.

### Nedd4 is not involved in the entry process of JEV

**Figure 3:**
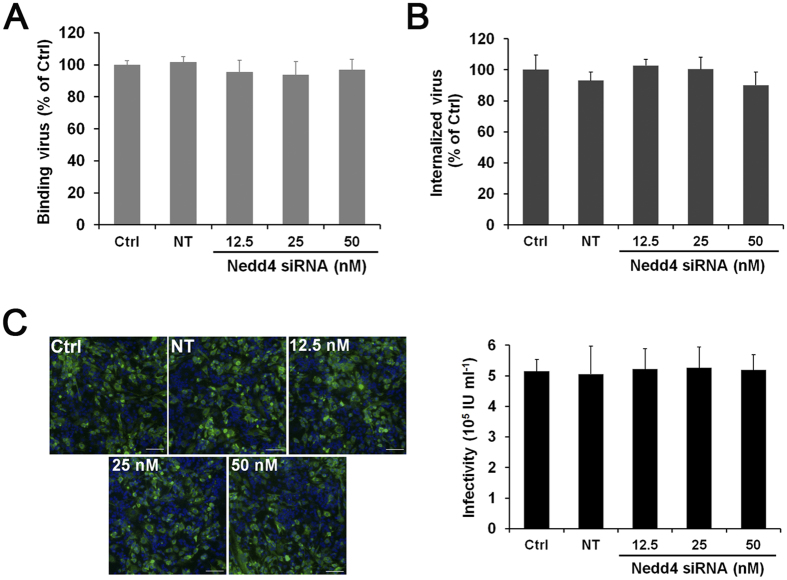
Nedd4 does not affect JEV entry. SK-N-SH cells were transfected with different concentrations of Nedd4-specific siRNA or the NT siRNA (50 nM) for 72 h to establish gene knockdown. (**A**) SK-N-SH cells were incubated with JEV (MOI = 10) at 4 °C for 1 h and then collected. The binding of JEV RNA was detected by RT-qPCR. (**B**) SK-N-SH cells were incubated with JEV (MOI = 10) at 4 °C for 1 h, and the cells were then transferred to 37 °C for another 1 h. A JEV internalization assay was performed, and JEV RNA was analysed by RT-qPCR. (**C**) SK-N-SH cells were infected with JEVpv, and the infectivity was assessed by identifying GFP-positive cells. The nuclei were stained with DAPI (blue). Images were obtained using a confocal microscope (left panel, scale bar = 50 μm) and quantitated using ImageJ software (right panel). The data are shown as the mean ± SD of three independent experiments.

To elucidate the effect of Nedd4 on the JEV life cycle, we first investigated whether Nedd4 knockdown affects JEV entry. We performed a viral binding assay to assess the effect of Nedd4 knockdown on attachment of JEV to SK-N-SH cells. As shown in Fig. 3A, the number of attached virions at the cell surface was not affected after Nedd4 knockdown, suggesting that Nedd4 does not participate in virus binding. Next, a viral internalization assay was used to examine the effect of Nedd4 knockdown on the internalization of JEV by measuring productive internalized virus particles. As shown in Fig. 3B, Nedd4 depletion resulted in no inhibitory effect on JEV internalization. In addition, JEV pseudotyped virus (JEVpv) system that can enter cells but not replicate was used. The single-life-cycle JEVpv contains JEV E glycoprotein and has been used to address the molecular mechanisms of JEV entry into cells. SK-N-SH cells transfected with Nedd4 siRNA were infected with JEVpv, and the infectivity was assessed by identifying GFP-positive cells. Similar to the JEV internalization assay, the infection of JEVpv was not impaired by the knockdown of Nedd4 (Fig. 3C). Collectively, these results clearly indicate that Nedd4 is not involved in the entry of JEV.

### Nedd4 regulates the replication of JEV and influences viral yields

**Figure 4:**
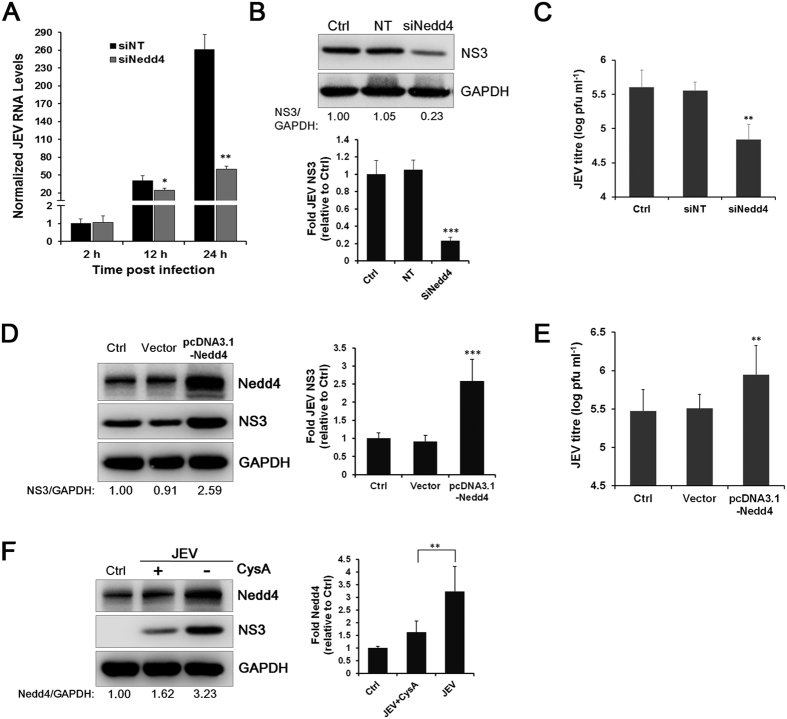
Nedd4 regulates the replication of JEV and influences viral yields. (**A**) Cells transfected with Nedd4 siRNA (50 nM) or the NT siRNA (50 nM) were infected with JEV (MOI = 1.0), and viral RNA levels were determined by RT-qPCR at 2 h, 12 h, and 24 h p.i. (**B**) JEV NS3 expression was evaluated by Western blot in Nedd4-depleted cells. GAPDH served as a control for equal sample loading. The level of NS3 was quantitated by densitometric analysis using ImageJ software and normalized to GAPDH. (**C**) The virus titres of the culture supernatants from Nedd4-depleted cells were determined by plaque forming unit assays. (**D**) Cells transfected with a plasmid expressing human Nedd4 or with an empty vector were infected with JEV (MOI = 1.0). Nedd4 and JEV NS3 expression was evaluated by Western blot. (**E**) The virus titres of the culture supernatants from Nedd4-overexpressed cells were determined by plaque forming unit assays. (**F**) Cells were infected with JEV (MOI = 1.0) for 1 h. The cells were then washed with 10% FBS DMEM and treated with CysA (5 μg/ml) in 10% FBS DMEM for 24 h. Nedd4 and JEV NS3 expression was evaluated by Western blot. The experiments were performed independently in triplicate. The data are shown as the mean ± SD. *, P < 0.05; **, P < 0.01; ***, P < 0.001.

Subsequently, the effect of Nedd4 on JEV replication was assessed. Cells transfected with Nedd4 siRNA were infected with JEV, and viral RNA levels were determined by RT-qPCR at the indicated times postinfection. As shown in Fig. 4A, viral RNA levels were essentially comparable at 2 h p.i., indicating similar virus uptake in both cell lines. However, JEV RNA levels were significantly reduced in Nedd4-depleted cells by approximately 4.33-fold compared with NT control cells at 24 h p.i. (Fig. 4A). This effect also manifested in a significant decrease in JEV NS3 expression in Nedd4-depleted cells (Fig. 4B), indicating that Nedd4 depletion significantly inhibited JEV replication. Furthermore, the virus titres of the culture supernatants from Nedd4-depleted cells were determined by plaque assay. As shown in Fig. 4C, knockdown of Nedd4 also decreased virus titres by approximately 5.87-fold at 24 h p.i. To test whether overexpression of Nedd4 can enhance JEV replication, SK-N-SH cells were transfected with a plasmid expressing human Nedd4 or with an empty vector as control. As shown in Fig. 4D, Nedd4 overexpression promoted JEV NS3 expression. The virus titres also increased by 2.89-fold 24 h p.i. after Nedd4 overexpression (Fig. 4E). In addition, when JEV replication was blocked by cyclosporin A (CysA), the up-regulation of Nedd4 by JEV infection was also inhibited (Fig. 4F), indicating that Nedd4 induction depends on viral replication. Taken together, these data suggest that Nedd4 participates in JEV replication and affects extracellular virus yields.

### Nedd4 promotes the replication of JEV by suppressing virus-induced autophagy

**Figure 5:**
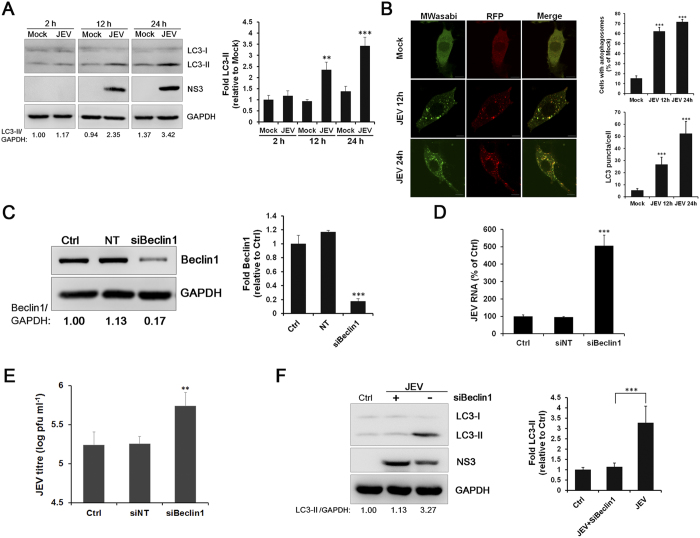
JEV replication is negatively regulated by autophagy in SK-N-SH cells. (**A**) SK-N-SH cells were infected with JEV (MOI = 1.0), and the cells were collected at 2 h, 12 h, and 24 h p.i. The protein levels of LC3-I, LC3-II and NS3 were evaluated by Western blot analysis. GAPDH served as a control for equal sample loading. The relative quantification of the detected signal was analysed using ImageJ software and normalized to GAPDH. (**B**) Representative fluorescent images and statistical results of punctate mTagRFP-mWasabi-LC3. Cells with autophagosomes were chosen from a pool of at least 10 images. Scale bar is 10 μm. (**C**) SK-N-SH cells were transfected with Beclin1 siRNA (50 nM) or the NT siRNA (50 nM). The level of Beclin1 protein was detected by Western blot. GAPDH was detected as a loading control. The relative quantification of the detected signal was analysed using ImageJ software and normalized to GAPDH. (**D** and **E**) The virus RNA was analysed by RT-qPCR (**D**), and the titres of the culture supernatants from Beclin1-depleted cells were determined by plaque forming unit assays (**E**). (**F**) SK-N-SH cells transfected with Beclin1 siRNA (50 nM) or the NT siRNA (50 nM) were infected with JEV (MOI = 1.0). The levels of LC3-I, LC3-II and NS3 were evaluated by Western blot analysis. GAPDH was detected as a loading control. The data are shown as the mean ± SD of three independent experiments. **, P < 0.01; ***, P < 0.001.

Nedd4-mediated protein ubiquitination controls multiple cellular processes. Previous studies have indicated that Nedd4 negatively regulates autophagy through Beclin1, a key regulator in autophagy, ubiquitination and degradation^22^. Recently, Nedd4 was shown to act as an LC3-interactive protein and directly regulates autophagy^19^. As autophagy has been shown to restrict the replication of JEV^23^, we suspected that Nedd4 may affect JEV replication by regulating autophagy. It was observed that JEV infection induced autophagy in SK-N-SH cells (Fig. 5A and B). JEV RNA levels and virus titers were significantly enhanced after Beclin1 knockdown (Fig. 5C, D and E). Moreover, in Beclin1-depleted cells, autophagy was significantly inhibited, while an increase in JEV NS3 expression was observed (Fig. 5F). These data suggest that JEV replication is negatively regulated by autophagy in SK-N-SH cells.

**Figure 6:**
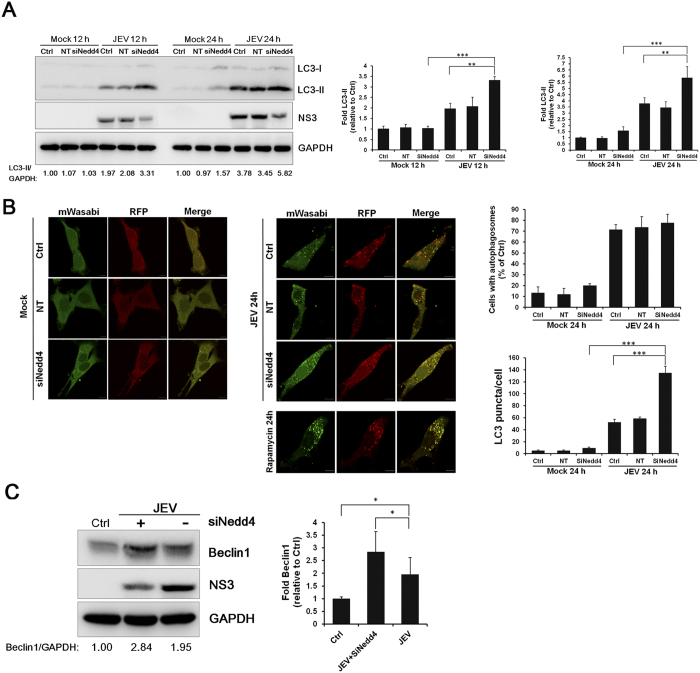
Nedd4 promotes the replication of JEV by suppressing virus-induced autophagy. (**A**) SK-N-SH cells transfected with Nedd4 siRNA (50 nM) or the NT siRNA (50 nM) were infected with JEV (MOI = 1.0), and the protein levels of LC3-I and LC3-II were determined by Western blot at 12 h and 24 h p.i. GAPDH served as a control for equal sample loading. The relative quantification of the detected signal was analysed using ImageJ software and normalized to GAPDH. (**B**) Representative fluorescent images and statistical results of punctate mTagRFP-mWasabi-LC3. Rapamycin (200 nM) treatment served as a positive control. Cells with autophagosomes were chosen from a pool of at least 10 images. Scale bar is 10 μm. (**C**) SK-N-SH cells transfected with Nedd4 siRNA (50 nM) or the NT siRNA (50 nM) were infected with JEV (MOI = 1.0). The levels of Beclin1 and NS3 were evaluated by Western blot analysis. GAPDH was detected as a loading control. The data are shown as the mean ± SD of three independent experiments. *, P < 0.05; **, P < 0.01; ***, P < 0.001.

Therefore, to investigate the possible relationship between Nedd4 and the autophagic pathway during JEV infection, Nedd4 was depleted by RNA interference, and then autophagosome accumulation was detected. Cells transfected with NT siRNA or Nedd4 siRNA were mock-treated or infected with JEV, and then the cell lysates were analysed by Western blot. Nedd4 depletion did not increase the LC3-II levels in mock-infected cells (Fig. 6A); however, the LC3-II levels were significantly enhanced in Nedd4 siRNA-transfected samples compared with NT-transfected cells after JEV infection (Fig. 6A), indicating that Nedd4 depletion leads to increased LC3-II accumulation. These observations were further confirmed by the detecting GFP-LC3 punctate distribution, which represented autophagosomes. As shown in Fig. 6B, there were 120 to 140 GFP-LC3 puncta per cell in Nedd4 siRNA-transfected cells compared with 40 to 60 in NT-transfected cells at 24 h p.i., indicating that Nedd4 depletion leads to enhanced autophagosome accumulation. As an inducer of autophagy, rapamycin treatment was used as a positive control, which resulted in a punctate GFP-LC3 pattern, representing autophagosomes (Fig. 6B). In addition, JEV infection led to an increase in the Beclin1 protein level in Nedd4-depleted cells (Fig. 6C). Taken together, these results suggest that Nedd4 negatively regulates viral-induced autophagy, which benefits JEV replication.

### Nedd4 shows no effect on JEV infection of non-neuronal cells

**Figure 7:**
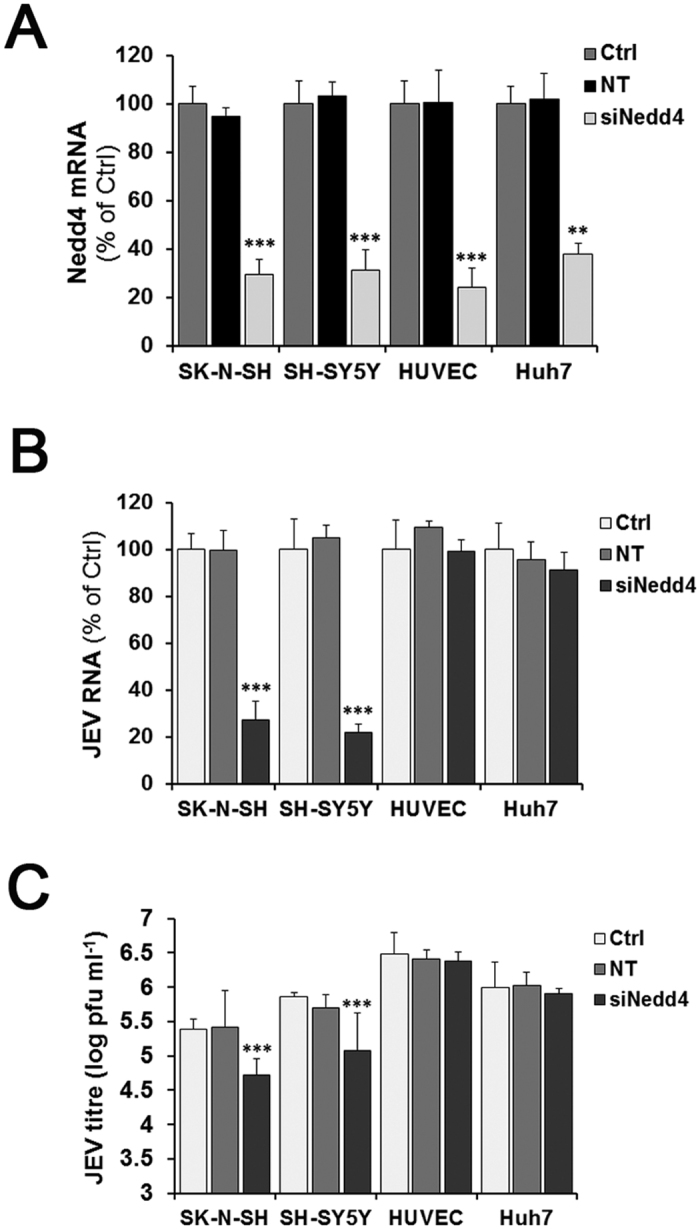
Nedd4 shows no effect on JEV infection of non-neuronal cells. SK-N-SH cells, SH-SY5Y cells, HUVECs, and Huh7 cells were separately transfected with Nedd4 siRNA (50 nM) or the NT siRNA (50 nM). (**A**) Nedd4 mRNA levels were determined by RT-qPCR at 72 h post-transfection. (**B**) Cells transfected with Nedd4 siRNA were infected with JEV (MOI = 1.0), and viral RNA levels were determined by RT-qPCR at 24 h p.i. (**C**) The virus titres of the culture supernatants from Nedd4-depleted cells were determined by plaque forming unit assays. The experiments were performed independently in triplicate. The data are shown as the mean ± SD. **, P < 0.01; ***, P < 0.001.

To determine whether Nedd4 plays a role in other human cell lines that are susceptible to JEV infection, we assessed the effect of Nedd4 depletion on JEV infection of human neuroblastoma SH-SY5Y cells, human umbilical vein endothelial cells (HUVECs) and human hepatocellular carcinoma Huh7 cells. These cells were transfected with Nedd4 siRNA and infected with JEV, and the viral RNA levels and Nedd4 mRNA levels were then determined by RT-qPCR at the indicated times. Down-regulation of Nedd4 by siRNA in these cells was confirmed by RT-qPCR (Fig. 7A). As shown in Fig. 7B, Nedd4 depletion significantly inhibited JEV infection of SK-N-SH cells and SH-SY5Y cells; however, no inhibitory effect was observed on JEV infection of Nedd4-depleted HUVECs or Huh7 cells. The similar results were observed when the virus titres of the culture supernatants from Nedd4-depleted cells were determined by plaque assays (Fig. 7C). These results indicate that Nedd4 has no effect on JEV infection of non-neuronal cells.

## Discussion

Successful viral infection requires hijacking and utilizing key cellular factors and pathways within host cells^24^. In this study, the important role of Nedd4, a ubiquitin ligase that belongs to the E3 class, in JEV infection of human SK-N-SH neuroblastoma cells was identified.

Viruses can utilize the host ubiquitin system at each stage of their life cycle, including cell entry, genome replication, budding and evasion of host innate immunity^25^. Here, we found that Nedd4, an important E3 ubiquitin ligase, participated in JEV infection. Although Nedd4 has been shown to be required for the infection of human immunodeficiency virus (HIV)^26^. Ebola virus^27^ and influenza A virus^28^, the roles of Nedd4 in viral infection of the CNS have not been well characterized yet. In our study, we found an unreported role of Nedd4 utilized by JEV to benefit its own replication in neuronal cells. It was observed that Nedd4 participated in JEV infection of neuronal cells but had no effect on JEV infection of non-neuronal cell lines, such as HUVECs and Huh7 cells. Considering that JEV is a neurotropic virus and that JEV infection leads to neuronal dysfunction and affects the normal development of neural stem cells^29^, the JEV infection-induced changes in Nedd4 expression and function may affect the pathogenesis of Japanese encephalitis.

Autophagy, an important cellular process that maintains cellular homeostasis, has been shown to play crucial roles in several types of viral infections^30^,^31^. However, autophagy plays different roles in different kinds of viral infections. For example, HSV-1 has evolved mechanisms to suppress cellular autophagy to allow its survival^32^, whereas poliovirus and Hepatitis C Virus (HCV) have evolved mechanisms to exploit autophagy to enhance viral replication^33^. Here, we show that JEV replication is negatively regulated by autophagy in human SK-N-SH neuroblastoma cells. JEV RNA levels, virus titers and JEV NS3 expression were significantly enhanced after Beclin1 knockdown in SK-N-SH cells, although it was found that JEV infection induced a significant increase in LC3-II form and Beclin1 upregulation. Our results are in line with a recent report that JEV-induced autophagy negatively regulates viral replication and protects cell viability in mouse neuro2a neuroblastoma cells^23^. These findings indicate that autophagy acts as an antiviral factor in response to JEV infection of neuronal cells.

Historically, the UPS and autophagy pathways were believed to be independent; however, accumulating studies have demonstrated that autophagy can be regulated by ubiquitination^34^. E3 ubiquitin ligases play a central role in the crosstalk between these two pathways^35^. To date, more than ten kinds of E3 ubiquitin ligases have been identified to interfere with autophagy^36^. The possible mechanism of this interaction is that E3 ubiquitin ligase can regulate the stability of certain autophagy proteins or upstream regulators, thereby affecting autophagic activity. Various studies have shown that Nedd4 is an important factor in autophagy regulation. Two earlier studies have shown that endoplasmic reticulum stress up-regulates Nedd4-2 to induce autophagy^20^ and that Nedd4 promotes cell proliferation and autophagy in the human prostate carcinoma cell line DU145a^37^, suggesting a positive role of Nedd4 in autophagy. However, it also has been shown that Nedd4 can polyubiquitinate Beclin1 to directly regulate autophagy^22^ or indirectly affect autophagy activity by regulating autophagy-related signal pathways, such as class III PI3-kinase^38^,^39^. These studies show a negative role of Nedd4 in autophagy. Moreover, a recent study shows that Nedd4 can directly interact with LC3 and is involved in autophagosomal biogenesis^19^. Here, we observed that Nedd4 suppresses autophagy in response to JEV infection in neuronal cells. In addition, we found that knockdown of Nedd4 led to an increase in the Beclin1 protein level, indicating that Nedd4 may promote Beclin1 degradation to reduce autophagy in SK-N-SH cells. This discrepancy in the effect of Nedd4 on autophagy between the studies might be due to differences in the context of autophagy between the different cell lines used in these experiments. Further studies should be pursued to clarify the role of Nedd4 in autophagy in different cells.

A recent study showed that the E3-ubiquitin ligase TRIM52, which belongs to the TRIM family of proteins, regulates JEV infection by directly interacting with NS2A of JEV and leading to NS2A degradation^40^. Whether Nedd4 can directly interact with JEV nonstructural proteins to affect autophagy activity and virus infection, and if these molecules and pathways are also exploited for replication by the emerging infectious neurotropic *Flaviviruses*, such as Zika virus and West Nile virus (WNV), remain interesting avenues to be explored in future studies.

In conclusion, our study identified Nedd4 as a novel host factor that is essential for JEV infection of human neuronal cells and demonstrated that Nedd4 promotes JEV replication by suppressing autophagy. Nedd4 down-regulation can significantly reduce JEV infectivity, thereby favouring the survival of neuronal cells. This work provides insight into how viruses exploit host proteins for their replication and identifies novel target for combating JEV-induced neurological diseases.

## Materials and Methods

### Cells and Viruses

The human neuroblastoma cell line SK-N-SH (ATCC, HTB-11), human neuroblastoma cell line SH-SY5Y (ATCC, CRL-2266), human umbilical vein endothelial cells (HUVECs) (ATCC, PCS-100-010), hepatocellular carcinoma cell line Huh7 and baby hamster kidney cell line BHK-21 (ATCC, CCL-10) were cultured in Dulbecco’s modified Eagle’s medium (DMEM) supplemented with 10% foetal bovine serum (FBS) (Invitrogen^TM^, Carlsbad, CA), 100 U/mL penicillin and 100 μg/mL streptomycin (Gibco^®^, Langley, OK). The JEV SA14 strain (GenBank accession no. U14163.1) was propagated in BHK-21 cells, and the virus titre assays were conducted in BHK-21 cells^41^.

### Antibodies, inhibitors and plasmids

Various commercially available antibodies were used in this study. Antibodies against LC3B (#3868), Beclin1 (#3495) and GAPDH (#2118) were obtained from Cell Signaling Technologies (Danvers, MA). The antibody against JEV NS3 (GTX125868) was from GeneTex (Irvine, CA). The antibody against Nedd4 (ab14592) was purchased from Abcam (Cambridge, MA). HRP-conjugated secondary antibodies against mouse (Cat #: 31430) or rabbit (Cat #: 31460) IgG, Alexa Fluor 488-conjugated goat anti-mouse IgG (Cat #: A32723) and Alexa Fluor 555-conjugated goat anti-rabbit IgG (Cat #: A32732) were purchased from Life Technologies (Invitrogen^TM^, Carlsbad, CA). Cyclosporin A (#S2286) and rapamycin (#S1039) were purchased from Selleck Chemicals (Houston, TX, USA). All of the plasmids used for JEV pseudotyped virus (JEVpv) generation were kindly provided by Yoshiharu Matsuura (Osaka University, Japan). A cDNA encoding the full-length human Nedd4 was cloned into the pcDNA3.1 vector. All constructs were confirmed by sequencing.

### Quantitative real-time PCR

Total RNA was extracted with TRIzol reagent (Takara, Tokyo, Japan) according to the manufacturer’s instructions. cDNA was synthesized using PrimeScript RT Master Mix (Takara) according to the protocol recommended by the manufacturer. mRNA levels were determined by quantitative real time PCR (RT-qPCR) using SYBR Premix Ex Taq (Takara). All reactions were performed in triplicate, and the mRNA level of the housekeeping gene glyceraldehyde-3-phosphate dehydrogenase (GAPDH) was used as an endogenous reference control. The primer set targeting the 3′-noncoding region of JEV included the forward primer 5′-CCCTCAGAACCGTCTCGGAA-3′ and reverse primer 5′-CTATTCCCAGGTGTCAATATGCTGT-3′. The primer set targeting Nedd4 included the forward primer 5′-GCATGTTTGCCATCCTCCCA-3′ and reverse primer 5′-AGCCAGGCTTGCAAGAATTAG-3′. The primer set targeting GAPDH included the forward primer 5′-TGGGCTACACTGAGCACCAG-3′ and reverse primer 5′-AAGTGGTCGTTGAGGGCAAT-3. The data were analysed relative to controls. All assays were performed on an ABI 7300 system (Applied Biosystems, Grand Island, NY).

### siRNA transfection

siRNA targeting Nedd4 (L-007178-00-0005) and Beclin1 (L-010552-00-0005) and a Non-Targeting siRNA (D-001810-10-05) were purchased from Dharmacon (Dharmacon, Pittsburgh, PA). All transfections were performed according to the manufacturer’s instructions using the transfection reagent FECT (Dharmacon). Cells were seeded at a density of 4 × 10^4^ cells/cm^2^ in 96-well plates. The following day, the cells were transfected with 12.5 nM, 25 nM, 50 nM, or 100 nM of siRNA mixed with OptiMEM (Invitrogen) and incubated at 37 °C for 72 h to ensure effective gene knockdown. Each siRNA transfection was performed in triplicate. Knockdown levels were monitored by RT-qPCR and Western blot at 72 h post-transfection.

### Cell transfection and transient expression

For plasmid overexpression, cells were transfected as described previously. Briefly, cells were seeded in 24-well tissue culture plates and grown overnight until they reached 75% confluency. Next, 0.8 μg of the plasmid construct was mixed with 50 μl of Opti-MEM (Invitrogen) for 5 min at room temperature. The mixture was then added to 50 μl of Opti-MEM containing 2 μl of Lipofectamine 2000 (Invitrogen) that had undergone similar incubation conditions. After a further incubation period of 20 min, the DNA-liposome complexes were added to the cells, which had been starved in Opti-MEM for 4 h before transfection. After 6 h of incubation at 37 °C, 1 ml of maintenance medium was added, and the mixture was incubated for an additional 48 h before virus infection.

### MTT cell viability assay

Cells (4 × 10^4^ cells/cm^2^) seeded in 96-well plates were transfected with increasing concentrations of siRNAs. After 72 h of incubation, the 3-(4,5-dimethylthiazol-2-yl)-2,5-diphenyltetrazolium bromide (MTT) assay was performed by adding 20 μl of 5 mg/ml MTT solution (Sigma-Aldrich, St. louis, MO) to the cells and incubating them for 4 h at 37 °C. The light absorbance of the solution was measured at 490 nm by a microplate reader (BioTek, Winooski, VT).

### JEV pseudotyped particle production and infection assay

JEVpv was generated as described previously^42^. To determine the infectivity of JEVpv, infected cells were identified as GFP-positive cells by fluorescence microscopy or using flow cytometry and expressed as infectious units (IU)/ml.

### JEV infection and virus titre determination

SK-N-SH cells in 96-well plates were transfected with the indicated siRNAs. The cells were infected with JEV at an MOI of 1.0 at 72 h post-transfection. A plaque forming unit (PFU) assay was used to measure the virus titres^41^. Confluent monolayers of BHK-21 cells were infected with 10-fold serially diluted JEV in 6-well plates. After 1 h of incubation, a 2% methylcellulose overlay was added. The plaques were visualized after being fixed with 10% formaldehyde and stained with crystal violet at 4 days post-infection. The amount of plaques was statistically analysed. All virus titres were expressed as PFU/millilitre (PFU/ml).

### Western blot

Cells were lysed in radioimmunoprecipitation assay (RIPA) buffer (Pierce, Rockford, IL). Equal amounts of protein were separated on 10% sodium dodecyl sulfate polyacrylamide gels (SDS-PAGE). The proteins were then transferred to polyvinylidene fluoride (PVDF) membranes (Bio-Rad, Hercules, CA). The membranes were blocked with 5% non-fat milk in 1 × Tris-buffered saline (TBS) with 1% Tween-20 (SCR, Shanghai, China) for 2 h at room temperature (RT). Then, the membranes were incubated with the indicated primary antibodies at 4 °C for 16 h. Detection was conducted by incubation with species-specific antibodies conjugated to HRP. Immunoreactive bands were detected with ECL Plus enhanced chemiluminescence Western blotting detection reagents (PerkinElmer Life Sciences, Waltham, MA).

### Immunofluorescence staining assay

SK-N-SH cells transfected with siRNAs were infected with JEV at an MOI of 1.0 and incubated for 2 h at 37 °C. At 48 h post-infection, the cells were fixed with 4% paraformaldehyde for 20 min and permeabilized with 0.1% Triton X-100 at RT for 10 min. Then, the cells were blocked in 5% bovine serum albumin (BSA) and incubated with an anti-JEV E mouse monoclonal antibody (a gift from The Fourth Military Medical University, Xi’an, China) at room temperature for 2 h. After being washed with phosphate-buffered saline (PBS) three times, the cells were stained with an Alexa Fluor 488 (AF 488)-conjugated anti-mouse antibody (Invitrogen) for 1 h. Nuclei were stained with 4′, 6′-diamidino-2-phenylindole (DAPI, Roche, UK) for 10 min.

### Measurement of virus internalization and binding

Viral internalization was detected as described previously^42^. SK-N-SH cells were seeded in 24-well plates (4.5 × 10^5^ cells/well). The following day, the cells were pretreated with JEV (MOI = 10) in 500 μl of DMEM at 4 °C for 1 h, after which the cells were moved to 37 °C. After 1 h of incubation, the cells were washed with PBS and treated with proteinase K (1 mg/ml) (Invitrogen) for 45 min at 4 °C to remove adsorbed but not internalized virus. The proteinase K was then inactivated with 2 mM phenylmethylsulfonyl fluoride (PMSF) in PBS with 3% bovine serum albumin (BSA). The cells were then washed three times with PBS, and 500 μl of TRIzol reagent (Invitrogen) was added per well for RNA isolation. The internalized viral RNA was analysed by RT-qPCR. For the JEV binding assay, cells were incubated with virus (MOI = 10) at 4 °C for 1 h, and the cells were then washed with PBS to remove unbounded virus. The total viral RNA was extracted and measured by RT-qPCR.

### Confocal microscopy

Cells seeded on coverslips were incubated with virus at 4 °C for 1 h, and then virus entry was initiated by moving the cells to 37 °C. At the indicated time points, the cells were fixed in 4% paraformaldehyde for 20 min and permeabilized with 0.1% Triton X-100 at RT. The cells were then blocked in 5% BSA and incubated with an Alexa Fluor 488 (AF 488)-conjugated anti-mouse antibody (Invitrogen) and Alexa Fluor 555 (AF 555)-conjugated anti-rabbit antibody (Invitrogen) for 2 h. After three washes with PBS, the cells were incubated with fluorochrome-conjugated secondary antibodies for 1 h. The cells were washed again three times with PBS, and the nuclei were stained with 4′,6-diamidino-2-phenyl-indole (DAPI, Roche) for 10 min. Images were obtained using a confocal laser scanning microscope (Zeiss LSM710 Meta; Carl Zeiss). The fluorescence intensity of the images was processed and quantified using ZEN Light Edition software (Carl Zeiss).

### Autophagic marker detection

The mTagRFP-mWasabi-LC3 plasmid was kindly provided by Prof. Jian Lin (Beijing Nuclear Magnetic Resonance Center; College of Chemistry and Molecular Engineering; Peking University; Peking, China)^43^. The mTagRFP-mWasabi-LC3 lentivirus was packaged using our own system consisting of the VSV-G, REV and MDL plasmids. SK-N-SH cells infected with the mTagRFP-mWasabi-LC3 lentivirus were transfected with Nedd4-specific siRNA or NT siRNA and then inoculated with JEV (MOI = 1.0) for 24 h. After two washes with PBS, the cells were fixed with 4% paraformaldehyde for 30 min. After another two washes with PBS, the cells were stained with DAPI and observed using a Zeiss LSM710 confocal microscope.

### Statistical analysis

All statistical analyses were performed using GraphPad Prism software Version 5 (GraphPad Software, La Jolla, CA). Statistical significance was determined using Student’s t test. All graphs represent the mean ± the standard error of the mean (SEM) unless stated otherwise, and statistical significance (P < 0.05) is indicated by an (*) asterisk. In addition, P < 0.01 and P < 0.001 are marked with two (**) and three (***) asterisks in the figures, respectively.

## Additional Information

**How to cite this article**: Xu, Q. *et al*. E3 Ubiquitin Ligase Nedd4 Promotes Japanese Encephalitis Virus Replication by Suppressing Autophagy in Human Neuroblastoma Cells. *Sci. Rep.* **7**, 45375; doi: 10.1038/srep45375 (2017).

**Publisher's note:** Springer Nature remains neutral with regard to jurisdictional claims in published maps and institutional affiliations.
